# Lowering positive margin rates at radical prostatectomy by color coding of biopsy specimens to permit individualized preservation of the neurovascular bundles: is it feasible? a pilot investigation

**DOI:** 10.1590/S1677-5538.IBJU.2017.0328

**Published:** 2018

**Authors:** Leslie A. Deane, Wei Phin Tan, Andrea Strong, Megan Lowe, Nency Antoine, Ritu Ghai, Shahid Ekbal

**Affiliations:** 1 Department of Urology, Rush University Medical Center, Chicago, IL, USA

**Keywords:** Robotic Surgical Procedures, Prostatectomy, Laparoscopy

## Abstract

**Objective:**

To evaluate whether color-coding of prostate core biopsy specimens aids in preservation of the neurovascular bundles from an oncological perspective.

**Materials and Methods:**

MRI guided transrectal ultrasound and biopsy of the prostate were performed in 51 consecutive patients suspected of being at high risk for harboring prostate cancer. Core specimens were labeled with blue dye at the deep aspect and red dye at the superficial peripheral aspect of the core. The distance from the tumor to the end of the dyed specimen was measured to determine if there was an area of normal tissue between the prostate capsule and tumor.

**Results:**

Of the 51 patients undergoing prostate biopsy, 30 (58.8%) were found to have cancer of the prostate: grade group 1 in 13.7%, 2 in 25.5%, 3 in 7.8%, 4 in 7.8% and 5 in 3.9% of the cohort. A total of 461 cores were analyzed in the cohort, of which 122 showed cancer. Five patients opted to undergo robotic assisted laparoscopic radical prostatectomy. No patients had a positive surgical margin (PSM) or extra prostatic extension (EPE) on radical prostatectomy if there was a margin of normal prostatic tissue seen between the dye and the tumor on prostate biopsy.

**Conclusion:**

Color-coding of prostate biopsy core specimens may assist in tailoring the approach for preservation of the neurovascular bundles without compromising early oncological efficacy. Further study is required to determine whether this simple modification of the prostate biopsy protocol is valuable in larger groups of patients.

## INTRODUCTION

Prostate cancer remains the most common solid organ malignancy in men globally with almost 181.000 new cases in the United States in 2016 (1). Albeit a topic of tremendous controversy, there have been significant advances in screening methods utilizing novel imaging, serum and urinary biomarkers. However, the diagnostic hallmark remains a transrectal ultrasound guided biopsy of the prostate for microscopic tissue and histopathological diagnosis (2, 3). A major advancement in this field was the introduction of multiparametric magnetic resonance imaging (mpMRI) of the prostate gland, conferring the ability to detect lesions with low, intermediate or high suspicion of being malignant (4). This development permitted targeted biopsies to be taken, focusing on these suspicious areas in addition to standard sextant template biopsies.

However, the ability of mpMRI to distinguish extracapsular extension (ECE) from organ-confined disease when a lesion appears to be in contact with the capsule remains poor and has very limited sensitivity (5). There are several surrogates indicative of ECE including tumor contact length and PIRADS score of the lesion in question, perineural invasion and Gleason score, but determination of extension at a microscopic level is not possible at this time (6, 7). A multitude of nomograms have been validated to estimate the risk of ECE, seminal vesicle invasion, lymph nodal involvement, organ confined disease and the clinical decision whether to spare or resect the neurovascular bundles (NVB) is largely based on these combinations and the judgment and experience of the surgeon.

Unless peri-prostatic fat, extra-prostatic neural tissue or rectal mucosa remain adherent to the biopsy core, it is difficult to orient the specimen into superficial versus deep regions. Currently, to our knowledge, no attempts have been made to localize the site of the cancer within individual biopsy cores nor to quantify its distance from the capsule. This could have significant implications for altering the surgical plan especially now that mpMRI is gaining wide acceptance. We sought to evaluate whether a system for color-coding transrectal mpMRI guided fusion prostate biopsy cores could reliably show where cancer was located in relation to periphery (i.e capsular aspect of the sample) in comparison to the deep aspect of the sample (i.e distant from the capsule).

## MATERIALS AND METHODS

We performed an Institution Review Board (IRB) approved retrospective analysis of a prospectively collected and maintained database consisting of all patients undergoing mpMRI transrectal ultrasound guided fusion prostate biopsy performed by a single urologist (LAD) for clinical features concerning the presence of prostate cancer. mpMRI of the prostate was performed using a Siemens MAGNETOM 3T machine (Munich, Germany) as previously described (8). Each MRI scan was reviewed and reported by an attending radiologist.

**Figure 1:**
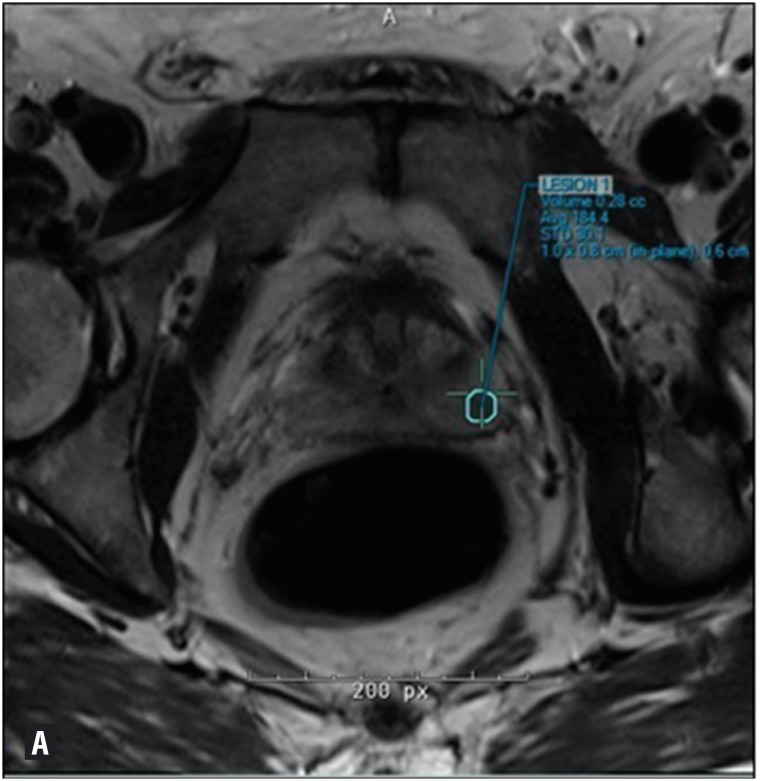
Volume hotspot on mpMRI of the prostate.

Each patient received prophylactic oral antibiotics (fluoroquinolone) prior to the procedure and an augmented regimen with an intramuscularly administered antibiotic if deemed high risk. Standard fleet enema was also administered the night before and at the morning of the procedure. All biopsies were performed transrectally using 3-dimensional modeling software (Invivo Corporation, Gainesville, FL, USA) and mpMRI / US fusion biopsy of the prostate was performed with an end-fire Philips iU22 transrectal ultrasound probe and sonographic system (Amsterdam, The Netherlands). A total of 5 cc of 0.5% bupivacaine was injected into the tissue in the angle of the seminal vesicle and prostate, and the periprostatic nerve plexus under transrectal ultrasonic guidance for intra-procedural patient comfort. Each biopsy sample was taken using an 18-gauge Bard Max-Core (Bard Medical Division, Covington, GA) and immediately handed to the circulating nurse or medical assistant. A minimum of 3 cores was taken from each suspicious lesion, largely dependent on the size of the volume hotspot on mpMRI (Figure-1). When obtaining the sample, a concerted effort was made to ensure that the needle insertion site was as close to a perpendicular entry across the capsule for peripheral zone lesions. The sample was placed onto a small square (3 × 4 cm) of Telfa (Kendall Telfa, Tyco Healthcare, Mansfield, MA) and verbally confirmed to be an intact core of adequate length. Pre-drawn insulin syringes with red dye (capsular aspect) and blue dye (deep aspect) (Davidson Tissue Marking System, Bradley Products Inc, Bloomington, MN) were used to color code each sample and the correct orientation was verbally affirmed prior to placing the sample into the specimen container, which was pre-labeled. A single drop of each color dye was applied to the core in its respective orientation. Each sample was allowed 30 seconds for the dye to dry prior to placing it into formalin in the specimen container and sent for pathological analysis.

**Figure 2:**
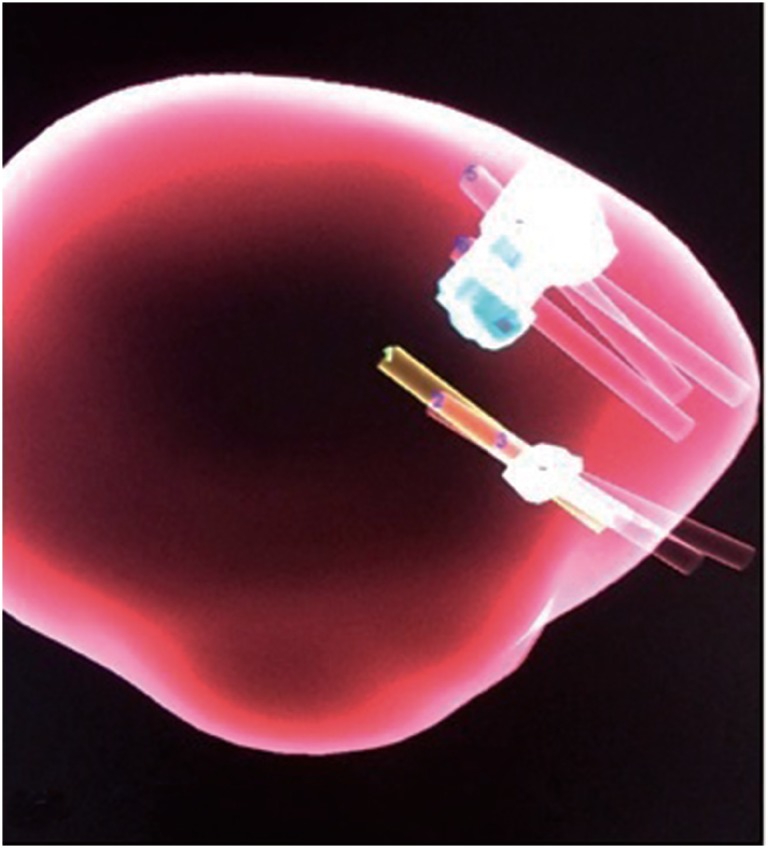
3-D image of prostate core acquisition.

Images from the mpMRI of the sample sites of each biopsy core were archived and saved for future reference and review (Figure-2). These were both 3-dimensional renderings and gray-scale images to demonstrate the path of the biopsy needle relative to each targeted lesion.

**Figure 3:**
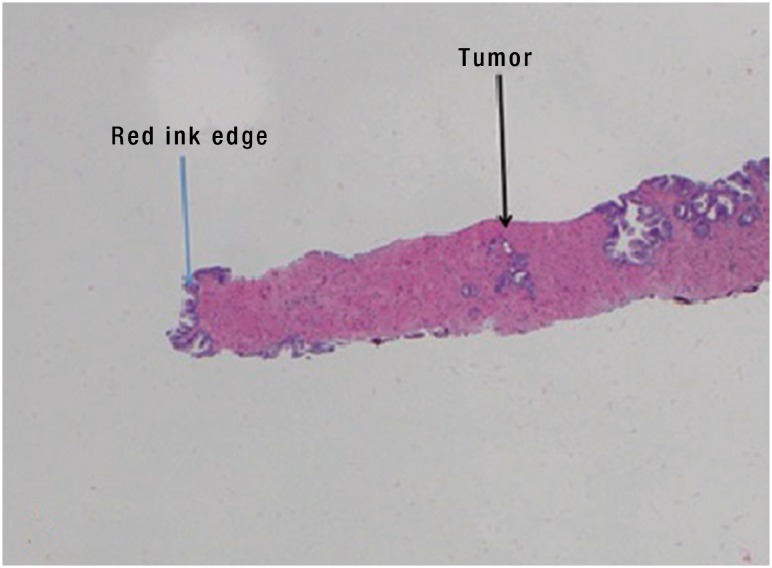
Relationship of cancer to peripheral margin.

Each prostate biopsy core was read by a team of genitourinary pathologists and reviewed with the treating urologist. Each sample was reported as either benign or malignant, the latter being assigned a Gleason Score and allocated to Grade Groups 1 through 5 (Figure-3).

**Figure 4:**
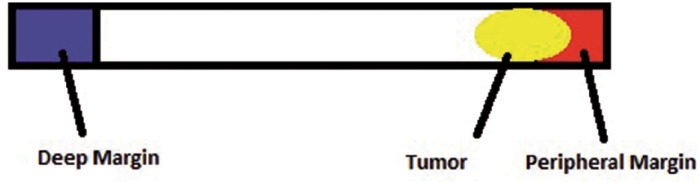
Example of tumor distance from end of dyed specimen.

If cancer was present, the distance from the tumor to the end of the dyed specimen was measured to determine if there was an area of normal tissue between the prostate capsule and tumor (Figure-4).

Patients who were found to have cancer were informed of all treatment options available. For patients electing to undergo robotic radical prostatectomy, the decision for nerve sparing was deemed based on established and accepted clinical standards and clinician oncologic judgment, irrespective of the distance of cancer from the dyed peripheral margin of the biopsy core. On all patients, on the involved side, complete neurovascular bundle excision was performed as per standard of care. On patients that did not have any cancer involving a particular side on mpMRI, only complete neurovascular bundle preservation was performed.

## RESULTS

**Figure 5:**
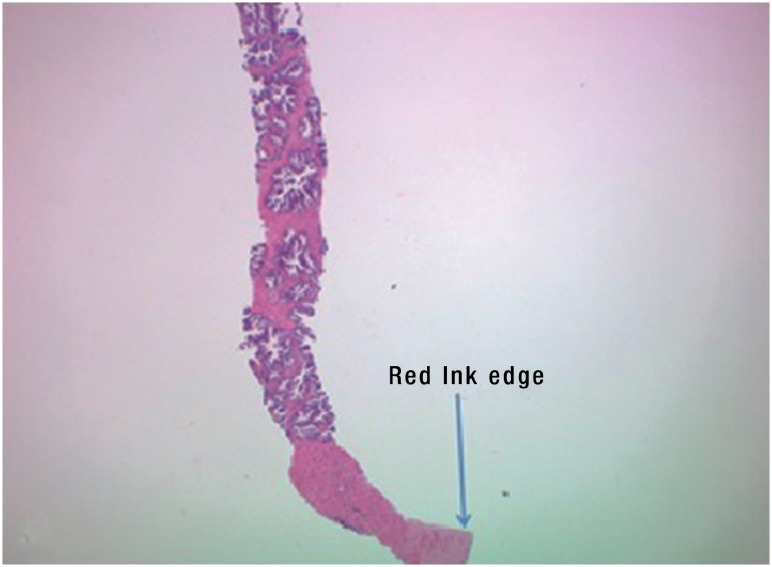
Distance from peripheral margin to malignant cells.

Of the 51 patients undergoing prostate biopsy, 30 (58.8%) were found to have cancer of the prostate. This was Grade Group 1 in 13.7%, 2 in 25.5%, 3 in 7.8%, 4 in 7.8% and 5 in 3.9% of the cohort. 21 patients (41.2%) did not have cancer on biopsy. A total of 461 cores were analyzed in the cohort, of which 122 showed cancer. The mean distance of tumor from the most superficial aspect of the red dye was 5.2 mm (Range 0 – 23 mm) (Table-1) (Figure-5). There were 10 cores with cancer < 1 mm from the red dye margin. 5 patients opted to undergo robotic assisted laparoscopic radical prostatectomy (RALP), 7 patients opted for radiation therapy, 1 patient switched provider and 17 patients were placed on active surveillance.

**Table 1:** Details of Cohort.

PATIENT CHARACTERISTICS		
Age, median (first/third quartile)		67 (62/70) years
**Ethnicity, n (%)**		
	Caucasian	17 (33)
	African American	28 (55)
	Hispanic	5 (10)
	Indian	1 (2)
PSA, median (first/third quartile)		6.7 (5.0-10) ng/mL
**Clinical Stage, n (%)**		
	No cancer	21 (41.2)
	T1c	27 (52.9)
	T2a	3 (5.9)
**Gleason Grade Group, n (%)**		
	No cancer	21 (41.2)
	1	7 (13.7)
	2	13 (25.5)
	3	4 (7.8)
	4	4 (7.8)
	5	2 (3.9)
Distance from red dye, median (first/third quartile)		4 (2-7) mm
PIRADS score (first/third quartile)		3 (3/4)

After reviewing these 5 patients in detail, all 5 patients had a lesion that appeared to be abutting or distorting the prostatic capsule (Table-2). Two patients had a lesion that was positive for cancer at the apex of the prostate and 3 patients had a lesion that was located in the lateral peripheral zone. Of the 2 patients that were positive for cancer at the apex, one patient had a tumor that distorted the prostatic capsule at the apex concerning of extracapsular extension seen on mpMRI while the other patient had a tumor abutting the prostate capsule on mpMRI. Both these patients had tumors involving the blue dye (deep) on the MRI / US guided fusion biopsy specimen and both these patients had ECE on the apical region on final pathology. This is because both these lesions were anterior and given the biopsy needle was activated from posterior to anterior, the blue dye represents the superficial aspect of the anterior prostate. Of the three patients who had tumors involving the red dye (superficial), one patient had tumor involving the edge of the red dye on the prostate biopsy specimen and he was found to have ECE on RALP. The other two patients that had a lesion in the lateral peripheral zone were found to have a margin between the red dye (superficial) and cancer. These two patients were subsequently found to be negative for ECE on radical prostatectomy, indicating that theoretically, the neurovascular bundle could have been spared.

**Table 2:** Pathological features of patients who underwent a radical prostatectomy.

Patient	Location of lesion on mpMRI	Distance of red dye from the edge of the biopsy specimen (mm)	Distance of blue dye from the edge of the biopsy specimen (mm)	Gleason Score on Biopsy	Gleason Score on Final Pathology	Extra Capsular Extension
1	Anterior Peripheral Zone		0; 0	4+3	3+4	Yes
2	Anterior Peripheral Zone		0; 0; 0; 0	4+5	4+5	Yes
3	Left Peripheral Zone	12; 9; 12; 1		4+4	4+3	No
4	Left Lateral Peripheral Zone	9; 8; 0		4+3	3+4	Yes
5	Left Lateral Peripheral Zone	3; 5; 5		3+4	3+4	No

**Figure 6:**
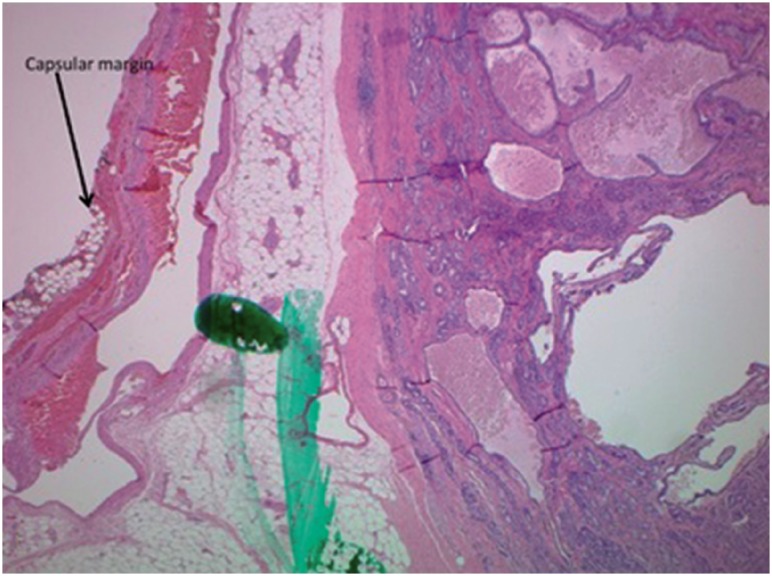
Capsular margin seen at radical prostatectomy - Distance from capsule to malignant cells demonstrated.

No patients had a positive surgical margin (PSM) or extra prostatic extension (EPE) on radical prostatectomy if there was a margin of normal prostatic tissue seen between the dye and the tumor on prostate biopsy (Figure-6). The first post-operative prostate specific antigen level PSA was undetectable (< 0.01 ng / mL) in all patients undergoing surgery.

The cost of the dye is $27.50 per 2 oz vial and this lasted throughout this entire initial pilot cohort. The total amount of dye used per biopsy was < 0.5 cc. The cost per box of insulin syringes is $17.38 and two syringes were used per patient at an additional cost of $0.35. As a result, the overall additional cost per biopsy was < $1.00, nominal when one considers the potential impact on surgical risk stratification and operative outcome, both oncologic and functional.

We have also confirmed that color coding the specimen does not alter the cellular architecture, nor does it affect the integrity of DNA / RNA material, thus having no potential for impacting genomic assessment of the tissue for further clinical risk analysis and stratification. There was 1 / 467 (0.002%) specimen core that was dyed incorrectly where the superficial and deep region of the core was inadvertently inverted. We realized this on pathology as there were fat cells and neural tissue seen on the specimen, thus confirming the true peripheral aspect of the core. The mpMRI images were reviewed in detail and showed the lesion in the central peripheral zone, and confirmed the trajectory of the biopsy needle.

## DISCUSSION

The technique of prostate biopsy continues to evolve and the specialty has progressed from finger guided, hand activated tru-cut sampling, through ultrasound guided sextant sampling with a trigger activated biopsy gun, and now mpMRI transrectal ultrasound fusion guided biopsy (9, 10). Despite significant advances in the ability to image the prostate, definitive imaging evidence of ECE on mpMRI remains elusive, unless gross tumor extension is observed. The accurate identification and reporting of biopsy specimens and their orientation is clinically relevant when it pertains to in office breast biopsy, frozen section in the operating room, and surgical specimens in all specialties (11-13). However, this concept has not been applied for prostate cancer biopsy specimens. This pilot study sought to assess the feasibility of color-coding prostate biopsy core samples with a view to determining how close the tumor was to the capsule cancer and if indeed the capsule was involved.

We obtained early evidence to show that a “normal tissue” interface between capsule (most peripheral aspect of the core dyed red) and malignant cells may be associated with a similar “normal tissue” interface at radical prostatectomy. This may have potential implications for surgeons in the decision-making algorithm regarding NVB sacrifice versus preservation.

Employing clinical risk nomograms such as the Memorial Sloan Kettering Prostate Risk Stratification Nomogram (MSKCC Nomogram), Cancer of the Prostate Risk Assessment tool (CAPRA), Partin tables, and the Stephenson Nomogram have characterized the risks of such entities as ECE and positive margins based on large cohorts of patients submitted to radical prostatectomy with pathologic and long-term clinical data and outcomes (14). In each of these nomograms, there is a defined risk of the pathological entity being present and similarly an inverse risk of the entity not being present. However, with the burgeoning field of genomic profiling of tumors and outcomes based on this information independent of clinical nomograms, we are aware that despite lesions having the same Gleason characteristics on biopsy and volume of cancer, they have biologically different behaviors. We are also cognizant that the nomograms in current use have not taken the spatial tumor location into consideration, as many were developed in an era pre-dating advanced imaging.

It is currently unclear as to what the peritumoral region on mpMRI is home to and what zone of mpMRI invisible tissue is positive surrounding the volume hot spot (15). One may argue that this is irrelevant as it relates to Gleason hot spot, however, and that any tumor outside of the hot spot (which represents high grade disease) that is not seen is likely to be lower grade disease. But we do not know this to be always true.

With this in mind, an ancillary pathologic correlate which is easily obtainable from the biopsy tissue, namely color-coding, and increased accuracy of tumor localization may be useful to incorporate into future nomograms which would also include mpMRI findings such as PIRADS score, overall tumor volume, tumor location, tumor-capsule contact length and a combination of single or multi-genomic data. Conceptually, this approach could also serve as a forewarning for the use of technologies such as confocal microscopy, optical coherence tomography and confocal laser endo-microscopy from the perspective of pre-emptively alerting the surgeon that malignant cells are known to be at a predetermined distance from the given plane of dissection (16-18). These factors may enable surgeons to take a more individualized approach to sparing of the NVB in a patient specific and disease-centric manner.

We acknowledge that our data is limited due to its small sample size and the few patients that went on to have radical prostatectomy as definitive therapy. It may also be best applied to posterior peripheral zone lesion but can also be applied to anterior zone lesions by careful study of the mpMRI lesion and the tract of the biopsy needles relatively to the anterior capsule (in this instance the blue dye would be the most peripheral as seen in 2 of our patients). Another limitation is this approach does not account for anteriorly based lesions and the propensity for a PSM at this site (19, 20). Handling this area at the time of prostatectomy remains a clinical / surgical judgment heavily reliant on imaging and intraoperative cues.

Despite the aforementioned shortcomings, we are enthused that this data represents an early proof of concept and have expanded the study to collect more patient data using this simple modification to the well-established biopsy protocol. It is our intention to incorporate that “normal tissue” interface concept into an algorithm with other nomograms, the goal being to predict the likelihood of cancer at the margin and ECE on a more individualized basis. This data may not be applicable in patients undergoing a transperineal biopsy.

## CONCLUSION

Color-coding of prostate biopsy samples obtained by mpMRI transrectal ultrasound and fusion biopsy is a simple adjunct to standard biopsy techniques, which may yield useful information regarding the proximity of malignant cells to the capsule and may provide useful information to complement surgical planning. Further study, with a larger patient cohort and pathological outcomes from radical prostatectomy, is needed to validate whether this approach may be beneficial when tailoring preservation of the NVB on an individualized basis.
